# Supplementary material for: Myogenin is an essential regulator of adult myofibre growth and muscle stem cell homeostasis
Source: eLife. 2020 Oct 1;9:e60445. doi: 10.7554/eLife.60445 (PMC7599067; doi:10.7554/eLife.60445)
Supplement: Supplementary file 1. [file elife-60445-supp1.docx]

Supplementary File 1

| **qPCR primers** | | |
| --- | --- | --- |
| **gene** | **Forward 5'-3'** | **Reverse 5'-3'** |
| *actinb2* | ATCCTTCTTGGGTATGGAAT | GACAATACAGTGTTGGCATA |
| *sep15* | CTCAAGTCCAAGCTTTTGTC | AGCCTCTCACATACTTGATT |
| *b2m* | TTGGCTCTCTCGAATAAAAC | CTTTCGGAGTGGAGACTTTC |
| *myod* | AACATTACAGTGGAGACTCTG | GTCATAGCTGTTCCGTCTTC |
| *myf5* | ATGGCCTCAGATGAATCAAA | CATTGTGCTAGCATTTTGTG |
| *mrf4* | CAGGAGAACCCAGATCATTC | GGACTCTGAAGACTCCAAC |
| *myog* | TCAGAAACACCCACAAACGCTCAC | GCAGGCCCAGGGGAGACACT |
| *pax7a* | CAAGAAAGATGACGATGACG | GTGCGATTACCTTTATCCC |
| *pax7b* | CGGGATACCAGTATAGTCAG | CATTCTTTGCCAGGTAATCC |
| *pax3a* | TTCCTTCAGTGAGTTCCATC | CGTTTCCACCAAATTTACAG |
| *pax3b* | CACTCACAATAACAACGCTA | ATGGAGTTATCAGTCCCATC |
| *myomaker* | GGACAACTTATTCACAGGGA | TCTGTGATTTTGACAAGCAG |
| *myomixer* | TCTGGTTGTCCGACTCTTCG | TTAAGAAGGCACAGGACGCA |
| *mef2d* | TCAAACAAGCTCTTCCAGTA | GTGCTCTTTCTTGTTCAGAG |
| *mylpfa* | TCCCTTTCTTGCTTTCTACC | GAAAACAATCCAATGTCCCC |
| *flnca* | CAAACACATACCAGGAAGTC | TCATTCTTCCTTCTCCTGTT |
| *mybpc1* | ATCCGTCCTACTCCAAATAA | ATAACCATTATCCGCTGAGT |
| *cdkn1a* | AAGGAAAACATCCCGAAAAC | GCTTGGTAGAAATCTGTGATG |
| *cdkn1ca* | TTCTTCAGTCCTCAGAAACA | AAGAAGTCTGTAATTTGCGG |
| *igf1* | GGCTTTTATTTCAGCAAACC | GTTGTGTGACCTTCTTGAAC |
| *igfr1a* | GATGTCTCCAGAGTCTTTGA | CATAAAACCACACCAAACGA |
| *igfr1b* | GTGTACATTCGCACTAAACC | TGAAGAATTAGCGTATGCAC |
| *myostatinb* | CAAGACACTGTGCAATAGAA | CATAGTCATATGAAGCGGTG |
| *tsc1a* | CATTAATGGCAGTCAGGAAG | GAAATGAATGAGTAAGGGCG |
| *tsc1b* | AAGAACATTTTCCACTCGAC | AAGACACAAGGTCCAATCAT |
| *tsc2* | AAACTAGAGTCTCAGTCCAG | GCATGACCACCTGATATAGA |
